# Psychische und körperliche Belastung im Rettungsdienst: Zusammenhang des arbeitsbezogenen Verhaltens und der Beanspruchungsfolgen

**DOI:** 10.1007/s00103-022-03584-1

**Published:** 2022-09-09

**Authors:** Irina Böckelmann, Beatrice Thielmann, Heiko Schumann

**Affiliations:** Bereich Arbeitsmedizin, Medizinische Fakultät, Otto-von-Guericke-Universität Magdeburg, Leipziger Str. 44, 39120 Magdeburg, Deutschland

**Keywords:** Einsatzkräfte, Arbeitsmedizin, Belastungsfaktoren, Arbeitsbezogenes Verhalten und Erleben, Präventionsstrategien, Emergency services, Occupational medicine, Stressors, Work-related behavior and experience, Preventive strategies

## Abstract

**Hintergrund:**

Einsatzkräfte im Rettungsdienst (RD) unterliegen zahlreichen arbeitsbezogenen Belastungsfaktoren. Diesen stehen verschiedene Ressourcen gegenüber, die der Belastung entgegenwirken können. Ziel der Arbeit war es, physische und psychische Belastungen im RD zu erheben sowie die Zusammenhänge zwischen dem arbeitsbezogenen Verhalten und den Folgen von (Fehl‑)Beanspruchung zu analysieren.

**Material und Methoden:**

An der Befragung im Jahr 2015 nahmen 276 Einsatzkräfte (39,3 ± 8,04 Jahre alt) teil. Sie beinhaltete berufsbezogene Fragen sowie den Fragebogen zur subjektiven Einschätzung der Belastungen am Arbeitsplatz nach Slesina, den Fragebogen zum arbeitsbezogenen Verhaltens- und Erlebensmuster (AVEM), den Erholungs-Belastungs-Fragebogen (EBF) und den Fragebogen für körperliche, psychische und soziale Symptome (KOEPS).

**Ergebnisse:**

Die von dem größten Teil der Befragten angegebenen Belastungen waren: ungünstige Körperhaltung, Heben/Tragen schwerer Lasten, körperliche Arbeit und Schichtarbeit. Am häufigsten fühlten sie sich durch Schichtarbeit belastet (76,9 % „oft“). Circa ein Drittel der Befragten zeigte in seinem arbeitsbezogenen Verhalten ein Risikomuster. In EBF und KOEPS traten keine Abweichungen vom Referenzbereich auf. Merkmale der emotionalen Einstellung gegenüber der Arbeit korrelierten positiv mit der Qualität der Erholung sowie negativ mit der Beanspruchung und gesundheitlichen Beschwerden. Das Perfektionsstreben, die offensive Problembewältigung und der berufliche Ehrgeiz waren nicht bzw. kaum mit den Beanspruchungsfolgen assoziiert.

**Diskussion:**

Die Gefährdungsbeurteilung und die Erfassung des arbeitsbezogenen Verhaltens von Einsatzkräften geben Ansatzpunkte für Präventionsmaßnahmen. Auf Grundlage von AVEM-Mustern können diese individuell angepasst werden.

## Abstract (en)

**Background:**

Ambulance workers are subject to a variety of work-related stress factors. These are counteracted by work-related resources. The aim of this work was to survey the mental and physical stress factors in emergency services personnel and to analyze the relationships between work-related behavior and stress consequences.

**Materials and methods:**

Two hundred and seventy-six emergency services personnel (39.3 ± 8.04 years old) participated. The survey included occupational questions as well as questionnaires for the subjective assessment of stress at work according to Slesina, work-related behavior and experience patterns (AVEM), recovery stress (EBF), and physical, psychological, and social symptoms (KOEPS).

**Results:**

The stresses reported by most respondents were awkward posture, lifting/carrying heavy loads, physical labor, and shift work. The highest rating for stress intensities was shift work (76.9% “often”). About one-third of the respondents showed a risk pattern of work-related behavior. KOEPS and EBF results were not abnormal. Behavioral traits that reflect job-related emotions correlated positively with recovery and negatively with strain and health complaints, which is consistent with theory. Striving for perfection, offensive problem solving, and professional ambition were not or hardly associated with the stress consequences.

**Discussion:**

The risk assessment and collection of work-related behavioral data of emergency services personnel with regard to coping with the jobʼs demands provide starting points for organizational and individual preventive measures. Based on AVEM templates, these can be customized.

## Hintergrund

Einsatzkräfte im Rettungsdienst (RD) werden in ihrem Arbeitsalltag einer großen Anzahl von aufgaben- und organisationsbezogenen Belastungsfaktoren ausgesetzt [1]. Aus den Rahmenbedingungen der Organisation können sich zahlreiche Arbeitsanforderungen (engl.: „job/work demands“) ergeben [2]. Die Arbeit ist gekennzeichnet durch wechselnde Einsatzorte, teilweise schlechte Witterungsbedingungen sowie durch Zeit‑, Verantwortungs- und Leistungsdruck. Belastungen können sich auch aus dem häufigen Erleben von schweren Unfällen und lebensbedrohlichen Erkrankungen, Erfahrungen von Misserfolg und den damit verbundenen Unsicherheiten, den Konsequenzen von Fehlern und dem häufig fehlenden Feedback über den weiteren Verlauf der Patient:innen ergeben. Zudem können Einsatzkräfte aufgrund der sozialen Kontrolle durch u. a. Familienangehörige oder Passanten, des Wartens auf den nächsten Einsatz, der Nachtschichten oder des erhöhten Infektionsrisikos Belastung empfinden [1, 3]. Dazu kommen die speziellen psychoemotionalen Belastungen, wie z. B. durch pädiatrische Einsätze, Einsätze im Familien- oder Bekanntenkreis oder unklare Lagen bei Unfall‑, Gewalt- und Vergiftungsgefährdungen [1]. Die belastenden Arbeitsbedingungen der Einsatzkräfte und die hohen Anforderungen nehmen Einfluss auf die psychische Stabilität, die Arbeitszufriedenheit und die Gesundheit [3, 4].

Während zu hohe Arbeitsanforderungen mit negativen Aspekten in Hinblick auf die psychische Gesundheit assoziiert werden [5, 6], hängen arbeitsbezogene Ressourcen (engl.: „job-/work-related resources“) mit positiven Aspekten, wie etwa Wohlbefinden [1, 7], zusammen. *Arbeitsbezogene Ressourcen,* wie z. B. Aufgabenvielfalt, Tätigkeits‑, Entscheidungs- und Handlungsspielräume, Qualifikationsnutzung, Entwicklungs- und Karrieremöglichkeiten, Partizipation und Weiterbildungsmöglichkeit, können die negativen Folgen von Arbeitsanforderungen auf die psychische Fehlbeanspruchung abpuffern [2, 8]. *Personale Ressourcen* im Arbeitsprozess (Kohärenzerleben, Selbstwirksamkeit, Stressbewältigungskompetenzen, internale Kontrollüberzeugungen, Zukunftsorientiertheit, Optimismus, fachliche Kompetenz) und *soziale Ressourcen* können ebenfalls positive Aspekte psychischer Gesundheit fördern [9, 10]. Zu den sozialen Ressourcen im Arbeitsprozess gehören soziale Netzwerke, positives Betriebsklima, positive Rückmeldungen, Anerkennung, Unterstützung durch Vorgesetzte, Kolleg:innen, Freund:innen und Familienangehörige sowie mitarbeiterorientiertes Vorgesetztenverhalten [10].

Neben der Fähigkeit zum aufgabenorientierten Handeln sowie der psychischen und körperlichen Belastbarkeit [11] sind insbesondere ein offener Erfahrungsaustausch, eine ausgeprägte Kollegialität und kollegiale Gesprächskultur bedeutsam für die Verarbeitung psychischer Belastung und für das Wohlbefinden der Einsatzkräfte [1, 12, 13]. Die Einsatzfrequenz und das Einsatzgebiet werden als entscheidende Prädiktoren für die subjektiv wahrgenommene Belastung und Beanspruchung der Einsatzkräfte betrachtet [14–16].

Theoretische Konzepte und Modelle zur Aufklärung des Zusammenhanges zwischen psychischer Belastung und psychischer Gesundheit sind z. B. das Belastungs-Beanspruchungs-Konzept nach Rohmert und Rutenfranz [17, 18], das transaktionale Stressmodell nach Lazarus [19], das Anforderungs-Kontroll-Modell (Job-Demand-Control-Model) von Karasek [20] und das Modell der beruflichen Gratifikationskrise (Effort-Reward-Imbalance-Model) von Siegrist [21]. Sie setzen unterschiedliche Schwerpunkte und ergänzen sich gegenseitig. Das Arbeitsanforderungs-Arbeitsressourcen-Modell (Job-Demands-Resources-Model) von Demerouti [22–24] bezieht neben Fehlbelastungen auch Ressourcen im Arbeitsprozess ein. Diese Modelle und Konzepte helfen, die Präventionsvorschläge auszuarbeiten.

Die zahlreichen Belastungen im RD können Erholungsprozesse der Einsatzkräfte beeinträchtigen und langfristig bei nicht ausreichenden arbeitsorganisationsbezogenen Ressourcen (z. B. Handlungs- und Entscheidungsspielräumen) und nicht vorhandenen personalen und sozialen Ressourcen in Erkrankungen resultieren [25]. Um die körperlich und emotional sehr anspruchsvollen Tätigkeiten im RD ausführen zu können, besteht eine Grundvoraussetzung in der physischen und psychischen Gesundheit der Einsatzkräfte. Eine erfolgversprechende Strategie zur Vermeidung von langfristigen Folgen psychischer Fehlbeanspruchung bzw. von Gesundheitsbeeinträchtigungen liegt in der gemeinsamen Betrachtung von aufgaben- und organisationsbezogenen Belastungsfaktoren, von Wegen zu deren Reduktion und von vorhandenen organisationalen, sozialen und personalen Ressourcen im Arbeitsprozess [1, 2].

Die Studienlage zeigt, dass die Belastungen und Beanspruchungen von Einsatzkräften sehr komplex sind, und verdeutlicht den weiteren Forschungsbedarf hinsichtlich Gesundheits- und Arbeitsschutzes im RD [1, 26, 27]. Insbesondere fehlen Untersuchungen zum arbeitsbezogenen Verhalten bei der Anforderungsbewältigung [14].

Vor diesem Hintergrund war es das Ziel der vorliegenden Arbeit, neben der Untersuchung der Belastungen von Einsatzkräften im RD die Zusammenhänge des arbeitsbezogenen Verhaltens mit den Beanspruchungsfolgen zu analysieren. Diese Fragestellungen sind von großer Bedeutung für die Arbeitsforschung und die Arbeitgeber im RD, die sich mit der gesetzlich verankerten Gefährdungsbeurteilung u. a. in Bezug auf eine psychische Belastung und psychosoziale Risiken auseinandersetzen müssen. Im modernen Arbeitsschutz stellt diese Gefährdungsbeurteilung ein zentrales Handlungsfeld dar [28]. Hier weist die Literatur noch große Lücken auf.

**Figure 1:**
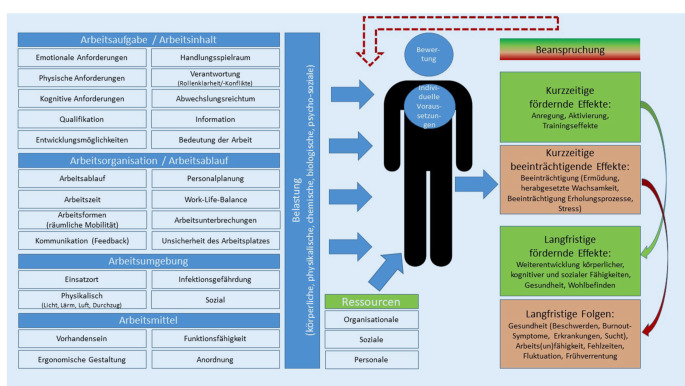

Die Grundlage dieser Studie bilden wichtige Merkmalsbereiche aus verschiedenen Gefährdungsbeurteilungen (wie bspw. Arbeitsaufgabe/-inhalt, Arbeitsorganisation/-ablauf, Arbeitsumgebung und Arbeitsmittel), die Erfassung der Belastungen und Anforderungen, die Bewertung dieser Belastungen nach dem transaktionalen Stressmodell, die Elemente aus dem Anforderungs-Kontroll-Modell (Handlungs- und Entscheidungsspielraum sowie die soziale Unterstützung durch Kolleg:innen und Vorgesetzte), die Komponente des Modells der beruflichen Gratifikationskrise (Anerkennung, Verausgabung) und Ressourcen in Anlehnung an das Job-Demands-Resources-Model (Abb. 1). Der Zusammenhang des arbeitsbezogenen Verhaltens mit der Beanspruchung und Erholung sowie gesundheitlichen Beschwerden werden genauer analysiert.

## Methoden

Die fragebogengestützte Querschnittserhebung zur Gesundheit von Einsatzkräften im RD wurde im Zeitraum 01/2015 bis 12/2015 im Rahmen einer größeren Studie durchgeführt. Ein Teil der weiteren Ergebnisse ist in einer Promotionsarbeit veröffentlicht [1].

### Probanden

Es wurden 850 Fragebögen an Einsatzkräfte aus dem RD der Hilfsorganisationen und der Berufsfeuerwehren im Raum Sachsen-Anhalt ausgeteilt. Die Rücklaufquote lag bei 32,5 % (*n* = 276), wobei die Gründe für die Nichtteilnahme unbekannt sind. Die Rekrutierung der Einsatzkräfte für diese Befragung erfolgte postalisch, Rettungsdienste und Rettungsschulen wurden angeschrieben und zur Teilnahme eingeladen.

Einschlusskriterien für die Teilnahme waren eine abgeschlossene Ausbildung im RD und eine hauptberufliche Tätigkeit als Rettungssanitäter:in, Rettungsassistent:in oder Notfallsanitäter:in. Zu den Ausschlusskriterien gehörten: wöchentliche Arbeitszeit unter 20 h und weniger als 3 Dienstjahre im RD.

An der Studie nahmen 276 Rettungskräfte teil: 261 Männer (94,6 %) und 15 Frauen (5,4 %). Das Durchschnittsalter betrug 39,3 ± 8,04 Jahre (23–62 Jahre). Unter den Befragten waren 70 (25,4 %) Einsatzkräfte aus dem RD der Berufsfeuerwehr und 206 (74,6 %) Personen aus dem RD der Hilfsorganisationen, wobei die Rettungsassistent:innen/Notfallsanitäter:innen mit 88,4 % (*n* = 244) und die Rettungssanitäter:innen mit 11,6 % (*n* = 32) vertreten waren.

### Messinstrumente und statistische Auswertung

Die Befragung beinhaltete soziodemografische und berufsbezogene Fragen, um die individuellen Voraussetzungen zu ermitteln, sowie 4 standardisierte Fragebögen.

Zur Beurteilung der Belastungssituation im RD wurde der *Fragebogen zur subjektiven Einschätzung der Belastungen am Arbeitsplatz nach Slesina* genutzt [29]. Die 47 verschiedenen Merkmale und Belastungsfaktoren unterteilen sich in 4 Bereiche: Arbeitsinhalt, Arbeitsorganisation, Körperhaltung und Arbeitsumweltfaktoren. Über die Frage: „Wie häufig oder wie stark trifft dieses Merkmal oder der Faktor auf Ihre Arbeit zu?“, wurde nach der subjektiven Bewertung der Einwirkintensität des jeweiligen Belastungsfaktors gefragt. Die Antwort erfolgte mittels einer 4‑Punkt-Skala („oft“, „mittel“, „selten“ und „nie“). Das Vorhandensein der Beanspruchung durch das jeweilige Merkmal bzw. den Belastungsfaktor wurde mit der Fragestellung: „Fühlen Sie sich selbst dadurch körperlich oder geistig belastet oder beansprucht?“, abgefragt, die jeweils mit „Ja“ oder „Nein“ beantwortet werden konnte. Dieser Fragebogen ermöglicht es, Aussagen zum eigenen Beanspruchungserleben zu treffen. Anforderungsintensität und anforderungsbedingtes Beanspruchungsempfinden können gegenübergestellt werden. Die Reliabilität der internen Konsistenz Cronbachs Alpha für die Bewertungsskalen ist 0,912 und für die Skalen des Vorhandenseins der Beanspruchung 0,943.

**Figure 2:**
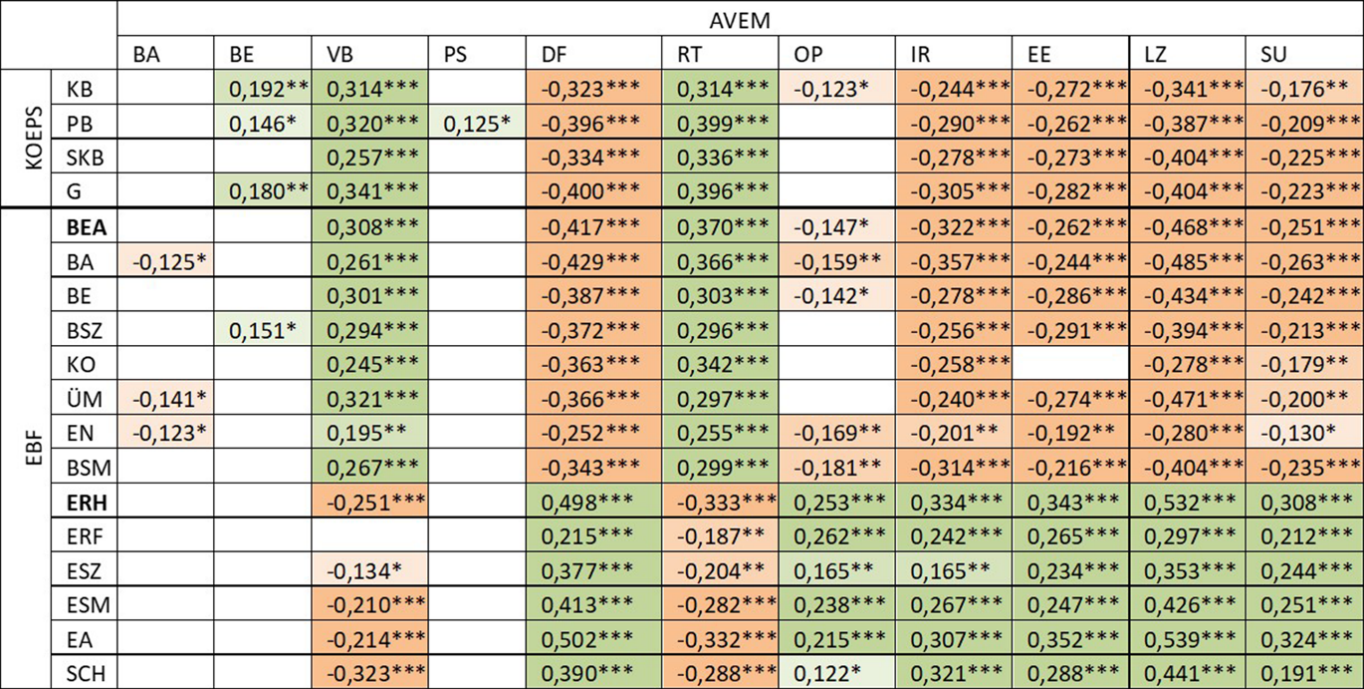

Mit dem *Fragebogen „Arbeitsbezogenes Verhaltens- und Erlebensmuster“ (AVEM)* von Schaarschmidt und Fischer [30] wurden Verhaltens- und Erlebensmerkmale in Bezug auf Arbeit und Beruf über 11 Dimensionen als personale und soziale Ressourcen (s. Ergebnisteil, Tab. 4) erfasst, die die inhaltlichen Bereiche des Arbeitsengagements, der psychischen Widerstandskraft und der emotionalen Einstellung gegenüber der Arbeit abbilden, wobei für diese Bereiche methodisch kein Score vorgesehen ist (Zuordnung s. Ergebnisteil, Abb. 2). Als Referenzbereich werden Stanine-Werte (abgeleitet von „standard scores with nine categories“) zwischen 4 und 6 beschrieben [31]. In AVEM ist die Reliabilität der internen Konsistenz Cronbachs Alpha 0,607. Den Befragten wurden anhand ihrer Ausprägungen in den Dimensionen gesundheitsgefährdende (Risikomuster A und B) bzw. gesundheitsfördernde (G und S) AVEM-Muster (s. Infobox 1, [31]) zugeordnet. Berücksichtigt wurden nur Muster mit einer „vollen“ Ausprägung (ein Muster von > 95 % Ausprägung), Muster mit einer „akzentuierten“ Ausprägung (ein Muster zwischen > 80 und ≤ 95 % Ausprägung) sowie Muster mit einer „tendenziellen“ Musterausprägung (ein Muster ≥ 60–≤ 80 %, kein zweites Muster mit ≥ 30 % Ausprägung).

Um den Grad von Erholung und Beanspruchung der Einsatzkräfte (als Outcomes) zu beurteilen, wurde der *Erholungs-Belastungs-Fragebogen (EBF)* nach Kallus [32] in der Kurzversion mit 25 Items verwendet. Dieses Verfahren beschreibt den gegenwärtigen psychischen sowie physischen Gesundheitszustand des Befragten. Hierzu werden die Häufigkeiten von Belastungs- und Erholungszuständen in den letzten 3 Tagen anhand einer Skala mit 7 Stufen (von „nie“ (0) bis „immerzu“ (6)) abgefragt. Die 25 Items wurden zu 12 Subskalen und 2 Hauptskalen (Beanspruchung und Erholung) zusammengefasst (Abb. 2). Hohe Ausprägungen der Werte sprechen für starke Beanspruchung/Belastungen (Referenzbereich 1,43–1,96) bzw. gute Erholungsaktivitäten (Referenzbereich 2,11–3,45; [32]). Die Reliabilität der internen Konsistenz Cronbachs Alpha der Skala Beanspruchung ist 0,919 bzw. der Erholung 0,837.

Anschließend wurden Symptome der letzten 4 Wochen mittels *Fragebogen für körperliche, psychische und soziale Symptome (KOEPS)* als negative langfristige Beanspruchungsfolgen (Outcomes) abgefragt [33]. Bei dem Fragebogen handelt es sich um eine Liste zur Selbstbeurteilung von Beschwerden, bestehend aus 60 Items, in der eine Dreiteilung von Symptomen (körperlich, psychisch und sozial) vorgenommen wird. Alle Symptome lassen sich zur „Gesamtbeeinträchtigung“ zusammenfassen. Die Aussagen werden mittels einer vierstufigen Skala von „traf nicht zu“ bis „traf sehr zu“ beantwortet. Je höher die Punktzahl bzw. der Stanine-Wert in der Auswertung, desto mehr Beschwerden wurden für den jeweiligen Bereich angegeben. Der Normwertbereich liegt zwischen 4 und 6. Im KOEPS ist die Reliabilität der internen Konsistenz Cronbachs Alpha 0,930.

Die ausgewählten Verfahren decken verschiedene Ebenen der Fragestellung ab. Entsprechend dem Vorgehen bei Gefährdungsanalysen bzgl. Psyche wurden die Ressourcen und (Fehl‑)Beanspruchungsfolgen deskriptiv analysiert.

Die *statistische Auswertung* wurde mithilfe des Statistikprogramms SPSS Statistics 28 (IBM Corp., Armonk, NY, USA) durchgeführt. Es erfolgten eine deskriptive Beschreibung der Gesamtstichprobe, die Erstellung von Kreuztabellen mit Chi-Quadrat nach Pearson und Korrelationsanalysen nach Spearman. Als Signifikanzniveau wurde eine Irrtumswahrscheinlichkeit von α = 5 % angenommen.

## Ergebnisse

### Berufsbezogene Daten

Die Dienstzeit der 276 befragten Einsatzkräfte betrug im Durchschnitt 12,9 ± 7,54 Jahre (3–35 Jahre). 59,8 % der Einsatzkräfte waren zum Erhebungszeitpunkt bereits länger als 8 Jahre im RD tätig. Ein Fünftel der befragten Einsatzkräfte gab eine Dienstzeit > 20 Jahre an.

Die Angaben zur durchschnittlichen Einsatzfrequenz, bezogen auf 12 h, variierten in Abhängigkeit von Einsatzgebiet (Größe, Lage) und Einsatzregion (Stadt, Land) zwischen 2 und 11 Einsätzen (im Durchschnitt 5,7 ± 1,86 Einsätze). In Bezug auf das hauptsächliche Einsatzgebiet gaben 195 (70,6 %) an, in dicht bis sehr dicht besiedelten Einsatzgebieten zu arbeiten und 81 (29,4 %) in dünn bis sehr dünn besiedelten Gebieten.

Die Teilnehmer:innen berichteten über eine wöchentliche Arbeitszeit von 51,4 ± 6,39 h (40–96 h/Woche). Eine Arbeitszeit von > 48 h/Woche gaben 139 (50,4 %) der Befragten an. Bei ihnen wird damit die in Deutschland gesetzlich zugelassene durchschnittliche Arbeitszeit überschritten.

### Subjektive Einschätzung der Belastungen am Arbeitsplatz

Zu den im Fragebogen nach Slesina oft genannten physischen Belastungsfaktoren (Tab. 1), durch die sich die Befragten selbst körperlich oder geistig belastet oder beansprucht fühlten, gehörten vor allem: ungünstige Körperhaltung (73,3 %), Heben (70,7 %) und Tragen (69,3 %) schwerer Lasten, körperliche Arbeit (65,8 %), Halten schwerer Lasten (59,6 %), Ziehen/Schieben schwerer Lasten (55,1 %). Die Intensität bzw. die Häufigkeit des Vorkommens dieser Faktoren am Arbeitsplatz lag zum größten Teil in den Bereichen „mittel“ und „oft“.

**Table 1:** 

Belastungsfaktor	„Wie häufig oder wie stark trifft dieses Merkmal oder der Faktor auf Ihre Arbeit zu?“		„Fühlen Sie sich selbst dadurch körperlich oder geistig belastet oder beansprucht?“	
Ungünstige Körperhaltung	*Nie*	1(0,4 %)	*Nein*	60(26,7 %)
	*Selten*	46(20,4 %)	*Ja*	165(73,3 %)
	*Mittel*	90(40,0 %)		
	*Oft*	88(39,1 %)		
Heben schwerer Lasten	*Nie*	1(0,4 %)	*Nein*	66(29,3 %)
	*Selten*	44(19,6 %)	*Ja*	159(70,7 %)
	*Mittel*	114(50,7 %)		
	*Oft*	66(29,3 %)		
Tragen schwerer Lasten	*Nie*	3(1,3 %)	*Nein*	69(30,7 %)
	*Selten*	39(17,3 %)	*Ja*	156(69,3 %)
	*Mittel*	114(50,7 %)		
	*Oft*	69(30,7 %)		
Körperliche Arbeit	*Nie*	0(0,0 %)	*Nein*	77(34,2 %)
	*Selten*	22(9,8 %)	*Ja*	148(65,8 %)
	*Mittel*	142(63,1 %)		
	*Oft*	61(27,1 %)		
Halten schwerer Lasten	*Nie*	7(3,1 %)	*Nein*	81(40,4 %)
	*Selten*	64(28,4 %)	*Ja*	134(59,6 %)
	*Mittel*	102(45,3 %)		
	*Oft*	52(23,1 %)		
Ziehen/Schieben schwerer Lasten	*Nie*	9(4,0 %)	*Nein*	101(44,9 %)
	*Selten*	69(30,7 %)	*Ja*	124(55,1 %)
	*Mittel*	98(43,6 %)		
	*Oft*	49(21,8 %)		
Zwangshaltung	*Nie*	19(8,4 %)	*Nein*	125(55,6 %)
	*Selten*	106(47,1 %)	*Ja*	100(44,4 %)
	*Mittel*	77(34,2 %)		
	*Oft*	23(10,2 %)		
Bewegungsmangel	*Nie*	17(7,6 %)	*Nein*	138(61,3 %)
	*Selten*	101(44,9 %)	*Ja*	87(38,7 %)
	*Mittel*	81(36,0 %)		
	*Oft*	26(11,6 %)		
Über-Kopf-Arbeit	*Nie*	43(19,1 %)	*Nein*	169(75,1 %)
	*Selten*	140(62,2 %)	*Ja*	56(24,9 %)
	*Mittel*	35(15,6 %)		
	*Oft*	7(3,1 %)		
Stehen	*Nie*	3(1,3 %)	*Nein*	177(78,7 %)
	*Selten*	55(24,4 %)	*Ja*	48(21,3 %)
	*Mittel*	125(55,6 %)		
	*Oft*	42(18,7 %)		
Sitzen	*Nie*	4(1,8 %)	*Nein*	184(81,8 %)
	*Selten*	49(21,8 %)	*Ja*	41(18,2 %)
	*Mittel*	134(59,6 %)		
	*Oft*	38(16,9 %)		
Handgeschicklichkeit	*Nie*	3(1,3 %)	*Nein*	186(82,7 %)
	*Selten*	42(18,7 %)	*Ja*	39(17,3 %)
	*Mittel*	104(46,2 %)		
	*Oft*	76(33,8 %)		
Vibrationen, Schwingung	*Nie*	44(19,6 %)	*Nein*	195(86,7 %)
	*Selten*	115(51,1 %)	*Ja*	30(13,3 %)
	*Mittel*	48(21,3 %)		
	*Oft*	18(8,0 %)		
Gehen	*Nie*	5(2,2 %)	*Nein*	203(90,2 %)
	*Selten*	49(21,8 %)	*Ja*	21(9,3 %)
	*Mittel*	113(50,2 %)		
	*Oft*	58(25,8 %)		

Organisatorische und psychische Faktoren (Tab. 2), durch die sich die Befragten selbst „körperlich oder geistig belastet oder beansprucht fühlten“, waren vor allem: Schichtarbeit (60,9 %), Termindruck (60,9 %), Wochenendarbeit (59,1 %) und Überstunden (56,9 %). Dabei wurde die Schichtarbeit am häufigsten als Belastung empfunden: 76,9 % antworteten hier mit „oft“.

**Table 2:** 

Belastungsfaktor	„Wie häufig oder wie stark trifft dieses Merkmal oder der Faktor auf Ihre Arbeit zu?“		„Fühlen Sie sich selbst dadurch körperlich oder geistig belastet oder beansprucht?“	
Schichtarbeit	*Nie*	6(2,7 %)	*Nein*	88(39,1 %)
	*Selten*	7(3,1 %)	*ja*	137(60,9 %)
	*Mittel*	39(17,3 %)		
	*Oft*	173(76,9 %)		
Termindruck	*Nie*	23(10,2 %)	*Nein*	88(39,1 %)
	*Selten*	89(39,6 %)	*Ja*	137(60,9 %)
	*Mittel*	69(30,7 %)		
	*Oft*	44(19,6 %)		
Wochenendarbeit	*Nie*	0(0,0 %)	*Nein*	92(40,9 %)
	*Selten*	18(8,0 %)	*Ja*	133(59,1 %)
	*Mittel*	100(44,4 %)		
	*Oft*	107(47,6 %)		
Überstunden	*Nie*	2(0,9 %)	*Nein*	97(43,1 %)
	*Selten*	77(34,2 %)	*Ja*	128(56,9 %)
	*Mittel*	94(41,8 %)		
	*Oft*	52(23,1 %)		
Ärger mit Vorgesetzten	*Nie*	37(16,4 %)	*Nein*	118(52,4 %)
	*Selten*	134(59,6 %)	*Ja*	107(47,6 %)
	*Mittel*	42(18,7 %)		
	*Oft*	12(5,3 %)		
Ärger mit Kolleg:innen	*Nie*	29(12,9 %)	*Nein*	125(55,6 %)
	*Selten*	149(66,2 %)	*Ja*	100(44,4 %)
	*Mittel*	39(17,3 %)		
	*Oft*	8(3,6 %)		
Zeitdruck	*Nie*	2(0,9 %)	*Nein*	130(57,8 %)
	*Selten*	53(23,6 %)	*Ja*	95(42,2 %)
	*Mittel*	99(44,0 %)		
	*Oft*	71(31,6 %)		
Verantwortung für Sicherheit, Gesundheit anderer	*Nie*	0(0 %)	*Nein*	137(60,9 %)
	*Selten*	26(11,6 %)	*Ja*	88(39,1 %)
	*Mittel*	77(34,2 %)		
	*Oft*	122(54,2 %)		
Leistungsdruck	*Nie*	15(6,7 %)	*Nein*	146(64,9 %)
	*Selten*	74(32,9 %)	*Ja*	79(35,1 %)
	*Mittel*	103(45,8 %)		
	*Oft*	33(14,7 %)		
Kontrolle durch Vorgesetzten	*Nie*	20(8,9 %)	*Nein*	153(68,0 %)
	*Selten*	123(54,7 %)	*Ja*	72(32,0 %)
	*Mittel*	68(30,2 %)		
	*Oft*	14(6,2 %)		
Konzentration	*Nie*	2(0,9 %)	*Nein*	156(69,3 %)
	*Selten*	33(14,7 %)	*Ja*	69(30,7 %)
	*Mittel*	94(41,8 %)		
	*Oft*	96(42,7 %)		
Selbstständiges Entscheiden	*Nie*	0(0 %)	*Nein*	161(71,6 %)
	*Selten*	24(10,7 %)	*Ja*	64(28,4 %)
	*Mittel*	100(44,4 %)		
	*Oft*	101(44,9 %)		
Nachdenken	*Nie*	2(0,9 %)	*Nein*	163(72,4 %)
	*Selten*	14(6,2 %)	*Ja*	62(27,6 %)
	*Mittel*	97(43,1 %)		
	*Oft*	112(49,8 %)		
Unterbrechungen durch Vorgesetzte	*Nie*	28(12,4 %)	*Nein*	169(75,1 %)
	*Selten*	143(63,6 %)	*Ja*	56(24,9 %)
	*Mittel*	50(22,2 %)		
	*Oft*	4(1,8 %)		
Selbstständige Arbeitsaufteilung	*Nie*	5(2,2 %)	*Nein*	183(81,3 %)
	*Selten*	53(23,6 %)	*Ja*	42(18,7 %)
	*Mittel*	78(34,7 %)		
	*Oft*	89(39,6 %)		

Der Umweltfaktor, der auf die meisten Befragten belastend oder beanspruchend wirkte, war Wärme/Hitze (41,3 %). 63,1 % der Befragten bewerteten die Häufigkeit bzw. Intensität dieses Belastungsfaktors mit „oft“ oder „mittel“ (Tab. 3). Beim Faktor Unfallrisiko lag die Häufigkeit/Intensität noch höher: 72,8 antworteten mit „mittel“ oder „oft“, belastet fühlten sich dadurch 37,8 %.

**Table 3:** 

Belastungsfaktor	„Wie häufig oder wie stark trifft dieses Merkmal oder der Faktor auf Ihre Arbeit zu?“		„Fühlen Sie sich selbst dadurch körperlich oder geistig belastet oder beansprucht?“	
Wärme/Hitze	*Nie*	5(2,2 %)	*Nein*	132(58,7 %)
	*Selten*	78(34,7 %)	*Ja*	93(41,3 %)
	*Mittel*	104(46,2 %)		
	*Oft*	38(16,9 %)		
Ungünstige Beleuchtung	*Nie*	10(4,4 %)	*Nein*	135(60,0 %)
	*Selten*	76(33,8 %)	*Ja*	90(40,0 %)
	*Mittel*	105(46,7 %)		
	*Oft*	34(15,1 %)		
Unfallrisiko	*Nie*	5(2,2 %)	*Nein*	140(62,2 %)
	*Selten*	56(24,9 %)	*Ja*	85(37,8 %)
	*Mittel*	91(40,4 %)		
	*Oft*	73(32,4 %)		
Nässe, Feuchtigkeit	*Nie*	9(4,0 %)	*Nein*	146(64,9 %)
	*Selten*	103(45,8 %)	*Ja*	79(35,1 %)
	*Mittel*	86(38,2 %)		
	*Oft*	27(12,0 %)		
Gerüche, Dämpfe	*Nie*	10(4,4 %)	*Nein*	148(65,8 %)
	*Selten*	88(39,1 %)	*Ja*	77(34,2 %)
	*Mittel*	84(37,3 %)		
	*Oft*	43(19,1 %)		
Lärm	*Nie*	8(3,6 %)	*Nein*	149(66,5 %)
	*Selten*	91(40,4 %)	*Ja*	75(33,5 %)
	*Mittel*	89(39,6 %)		
	*Oft*	37(16,4 %)		
Zugluft	*Nie*	18(8,0 %)	*Nein*	163(72,4 %)
	*Selten*	110(48,9 %)	*Ja*	62(27,6 %)
	*Mittel*	78(34,7 %)		
	*Oft*	19(8,4 %)		
Chemische Stoffe	*Nie*	22(9,8 %)	*Nein*	163(72,4 %)
	*Selten*	125(55,6 %)	*Ja*	62(27,6 %)
	*Mittel*	55(24,4 %)		
	*Oft*	23(10,2 %)		
Staub, Schmutz	*Nie*	19(8,4 %)	*Nein*	167(74,2 %)
	*Selten*	111(49,3 %)	*Ja*	58(25,8 %)
	*Mittel*	68(30,2 %)		
	*Oft*	27(12,0 %)		

### Arbeitsbezogenes Verhalten

Die Ausprägungen der einzelnen AVEM-Dimensionen in der Gesamtstichprobe sind in der Tab. 4 dargestellt. Die höchste Ausprägung war bei der Distanzierungsfähigkeit nachzuweisen (6,3 ± 1,54), die niedrigste bei der Dimension Erfolgserleben im Beruf (3,8 ± 1,67); die beiden Werte liegen außerhalb des Referenzbereichs. Alle weiteren Dimensionen sind dem Referenzbereich (4–6) einzuordnen.

**Table 4:** 

Merkmal	MW ± SD	(Min.–Max.)	Referenzbereich
**AVEM**			
Subjektive Bedeutung	4,5 ± 1,69	(1–9)	4–6
Beruflicher Ehrgeiz	5,5 ± 1,81	(1–9)	4–6
Verausgabungsbereitschaft	5,0 ± 1,66	(1–9)	4–6
Perfektionsstreben	4,8 ± 1,53	(1–9)	4–6
Distanzierungsfähigkeit	6,3 ± 1,54	(1–9)	4–6
Resignationstendenz bei Misserfolgen	4,2 ± 1,69	(1–8)	4–6
Offensive Problembewältigung	4,5 ± 1,78	(1–9)	4–6
Innere Ruhe und Ausgeglichenheit	5,5 ± 1,54	(2–9)	4–6
Erfolgserleben im Beruf	3,8 ± 1,67	(1–8)	4–6
Lebenszufriedenheit	4,1 ± 1,70	(1–8)	4–6
Erleben sozialer Unterstützung	4,7 ± 1,79	(1–8)	4–6
**KOEPS**			
Körperliche Beeinträchtigung	4,6 ± 1,69	(2–9)	4–6
Psychische Beeinträchtigung	4,5 ± 1,70	(1–9)	4–6
Sozialkommunikative Beeinträchtigung	4,3 ± 1,38	(3–9)	4–6
*Gesamtbeeinträchtigungen*	4,3 ± 1,82	(1–9)	4–6
**EBF**			
*Beanspruchung*	1,8 ± 0,93	(0,1–4,9)	1,43–1,96
Allg. Belastung, Niedergeschlagenheit	1,5 ± 1,16	(0–6)	0,50–2,04
Emotionale Belastung	1,8 ± 1,03	(0–5,5)	0,55–2,25
Soziale Spannungen	1,8 ± 1,16	(0–6)	0,61–2,11
Ungelöste Konflikte, Erfolglosigkeit	2,2 ± 1,21	(0–6)	0,95–2,67
Übermüdung, Zeitdruck	2,0 ± 1,22	(0–5,5)	0,88–2,90
Energielosigkeit, Unkonzentriertheit	1,8 ± 0,97	(0–4,5)	0,88–2,38
Körperliche Beschwerden	1,4 ± 1,05	(0–5)	0,41–1,81
*Erholung*	3,0 ± 0,92	(0,7–5,4)	2,11–3,45
Erfolg, Leistungsfähigkeit	2,3 ± 1,01	(0–5,5)	1,98–3,76
Erholung im sozialen Bereich	2,9 ± 1,22	(0,5–6)	2,06–4,00
Körperliche Erholung	3,0 ± 1,09	(0,5–5,5)	2,51–4,33
Allg. Erholung, Wohlbefinden	3,4 ± 1,16	(1–6)	2,78–4,58
Erholsamer Schlaf	3,4 ± 1,36	(0–6)	0,95–2,93

*AVEM* Fragebogen „Arbeitsbezogenes Verhaltens- und Erlebensmuster“*, KOEPS* Fragebogen für körperliche, psychische und soziale Symptome*, EBF* Erholungs-Belastungs-Fragebogen*, MW* Mittelwert*, SD* Standardabweichung*, Stanine*: „standard scores with nine categories“

Von den insgesamt 276 befragten Einsatzkräften konnten 205 (74,3 %) einem der 4 AVEM-Muster entweder mit einer „vollen“ (14,5 %, *n* = 40), einer „akzentuierten“ (30,4 % *n* = 84) oder einer „tendenziellen“ (29,4 %, *n* = 81) Ausprägung zugeordnet werden. Dabei wiesen 40 Befragte das Risikomuster A (19 %), 27 das Risikomuster B (13 %), 63 das Muster G (31 %) und 75 das Muster S (37 %) auf (vgl. Infobox 1). 51 Einsatzkräfte mit einer Musterkombination sowie 20 mit nicht zuzuordnenden Mustern wurden aus der weiteren Analyse ausgeschlossen.

### Beanspruchungsfolgen

Mit dem EBF-Fragebogen wurde der aktuelle Grad der Erholung und Beanspruchung ermittelt. Alle Subskalen innerhalb der Hauptskalen Beanspruchung und Erholung befanden sich im Durchschnitt innerhalb des Referenzbereiches (Tab. 4).

Die Ausprägungen der KOEPS-Dimensionen innerhalb der Gesamtstichprobe lagen zwischen 4,3 (sozialkommunikative Beschwerden) und 4,6 (körperliche Beeinträchtigungen) und somit noch im Normbereich (Tab. 4).

### Zusammenhänge zwischen dem arbeitsbezogenen Verhalten und Beanspruchungsfolgen

Zwischen den AVEM-Dimensionen und den KOEPS-Skalen sowie EBF-Subskalen/Hauptskalen bestanden positive und negative Assoziationen (Abb. 2).

Die AVEM-Dimension „Perfektionsstreben“ wies keine Assoziationen zu jeglichen EBF-Subskalen und zu körperlichen, sozialkommunikativen und Gesamtbeschwerden auf. „Subjektive Bedeutsamkeit der Arbeit“ und „beruflicher Ehrgeiz“ waren auch kaum mit einer Subskala der Beanspruchung oder Erholung assoziiert. „Verausgabungsbereitschaft“ und „Resignationstendenz bei Misserfolgen“ hingen moderat positiv und „ausgeprägte Lebenszufriedenheit“ moderat negativ mit den körperlichen, psychischen und sozialen Beschwerden zusammen. Distanzierungsfähigkeit, innere Ruhe und Ausgeglichenheit, Erfolgserleben im Beruf, Lebenszufriedenheit sowie Erleben sozialer Unterstützung korrelierten mit weniger Beschwerden und einer geringeren Beanspruchung.

## Diskussion

### Alter und Geschlechterverteilung

Die Altersstruktur der hier untersuchten Einsatzkräfte ist vergleichbar mit jener in anderen Studien und für Deutschland repräsentativ [1]. Die hier vorliegende Studie stellt ein deutliches Ungleichgewicht zwischen dem Anteil von Frauen (5,4 %) und Männern (94,6 %) fest. Zwar stieg laut Gesundheitsberichterstattung des Bundes der Frauenanteil im RD seit 2000 um knapp 8 % auf 30,5 % im Jahr 2015 an [34], jedoch zeigen auch andere nationale Studien hier geringere Anteile (zwischen 5 % und 11,4 %; [2, 15, 35]). In Studien, in denen der RD der Berufsfeuerwehren einen größeren Teil einnimmt, ist der Anteil der Frauen sogar noch geringer [36, 37].

### Belastung und Erholung

Auf der Grundlage der Ergebnisse aus dem Slesina-Fragebogen zu Belastungen von Einsatzkräften im RD und der Bewertung dieser Belastungen wird deutlich, dass in diesem Beruf zahlreiche physische und psychische Belastungsfaktoren in unterschiedlichem Maße vorkommen. Die ungünstige Körperhaltung war mit 73,3 % der am häufigsten genannte Belastungsfaktor. Die subjektive Bewertung der Einwirkintensität lag bei der Schichtarbeit am höchsten. Die negativen Folgen von Schichtarbeit auf das Schlaf- und Gesundheitsverhalten sind bekannt [38, 39].

Finden Erholungsprozesse nicht in angemessener Weise statt, können Stressoren Krankheiten verursachen [40]. Auch Schwierigkeiten, sich zu erholen, können als ein Vorzeichen für spätere Gesundheitseinschränkungen [41] gesehen werden. Eine gute und ausreichende Erholung ist unabdingbar, um die bei der Arbeit entstehenden beeinträchtigenden Beanspruchungsfolgen abzubauen [41]. Sie beugt Ermüdungs- und Erschöpfungssymptomen vor und sichert vermutlich gleichzeitig die Leistungsfähigkeit der Einsatzkraft. Die Werte für Erholung sind in der hier befragten Stichprobe zwar nicht auffällig, jedoch lag ihre durchschnittliche Ausprägung an der unteren Grenze des Referenzbereiches. Der Zusammenhang der Lebenszufriedenheit mit der Erholung lässt darauf schließen, dass Einsatzkräfte mit einer geringeren Ausprägung der Lebenszufriedenheit (Risikomuster A und B) in Präventionsprogrammen stärker berücksichtigt werden sollten.

### Ressourcen

Die organisationalen, sozialen und personalen Ressourcen haben für den Umgang mit belastenden Situationen im Arbeitsalltag des RD für die Abwendung von Fehlbeanspruchungen und die Verhütung arbeitsbedingter Erkrankungen sowie für die Gesundheitsförderung einen besonderen Stellenwert.

Bekannt ist, dass der Handlungs- und Entscheidungsspielraum als organisationale Ressource dient und Belastungen entgegenwirken kann [42, 43]. Das selbstständige Entscheiden und die selbstständige Arbeitsaufteilung gehören zwar zu den Merkmalen der Arbeit, von denen sich die Einsatzkräfte selten belastet oder beansprucht fühlten, jedoch wird die Häufigkeit/Intensität des Einwirkens dieser Faktoren von fast jedem Befragten als „oft“ oder „mittel“ beurteilt (89,3 % bzw. 74,3 %). Auch die Kontrolle durch Führungskräfte im RD wird von 32,0 % als belastend empfunden.

Eine wichtige vorbeugende Rolle hinsichtlich der Entstehung psychischer Beeinträchtigungen und Erkrankungen infolge von Belastungen am Arbeitsplatz spielt das Vorhandensein ausreichender sozialer Ressourcen, v. a. die soziale Unterstützung durch Kolleg:innen und Vorgesetzte [10, 14]. Dies trifft auch auf den RD zu [11]. Hier sind Zusammenhänge arbeitsbezogener Verhaltensmerkmale wie „innere Ruhe“ und „Ausgeglichenheit“, „Erfolgserleben im Beruf“, „Lebenszufriedenheit“ und „Erleben sozialer Unterstützung“ zu verzeichnen: positive mit der Erholung und negative mit Beanspruchung und Beschwerden. Im hier eingesetzten EBF-Verfahren wird Erholung unter dem Gesichtspunkt der Wechselbeziehung zwischen Arten der Belastungen und Beanspruchungen und Arten von Erholungsaktivitäten gesehen [32]. Das Erleben sozialer Unterstützung wird als ein „psychologischer Schutzfaktor“ in kritischen Situationen und als ein unmittelbarer Ausdruck von Wohlbefinden und damit psychischer Gesundheit gesehen [30].

### Verhaltensmerkmale, Beanspruchungsfolgen und Ansätze der Prävention und Gesundheitsförderung

Fast alle individuellen arbeitsbezogenen Verhaltensmerkmale der befragten Einsatzkräfte korrelieren mit der Beanspruchung, der Erholung und den gesundheitlichen Beschwerden. Die Beanspruchungsfolgen sind überraschenderweise jedoch nicht mit der subjektiven Bedeutsamkeit der Arbeit, dem beruflichen Ehrgeiz und dem Perfektionsstreben assoziiert. Diese 3 Verhaltensmerkmale scheinen für die Entstehung der negativen Beanspruchungsfolgen nicht relevant zu sein. Eine stärkere Verausgabung der Einsatzkräfte hängt theoriekonform mit einer höheren Beanspruchung und vermehrten körperlichen, psychischen und sozialkommunikativen Beschwerden sowie mit einer geringeren Erholung zusammen.

Eine hohe Distanzierungsfähigkeit ist dagegen mit geringerer Beanspruchung und seltenen Beschwerden sowie besserer Erholung der befragten Einsatzkräfte assoziiert. In der hier untersuchten Stichprobe war die Distanzierungsfähigkeit stärker als im Referenzbereich ausgeprägt. Das ist evtl. auch der Grund, warum die Erholungsprozesse noch nicht beeinträchtigt waren. Die Distanzierungsfähigkeit wird hier als wichtige Ressource der Einsatzkräfte angesehen.

Die gesundheitlichen Beschwerden liegen im KOEPS zwar im Normbereich, die Lebenszufriedenheit korreliert jedoch negativ mit sozialkommunikativen Beschwerden, was auch hypothetisch vermutet wurde. Präventionsvorschläge und gesundheitsförderliche Maßnahmen sollten daher insbesondere für solche Einsatzkräfte erarbeitet werden, bei denen eine geringere Ausprägung der Lebenszufriedenheit (Risikomuster A und B) vorliegt. Das Verhaltensmerkmal „offensive Problembewältigung“ korreliert positiv mit der Erholung. Das „Erfolgserleben im Beruf“ liegt in dieser Stichprobe etwas unterhalb des Referenzbereichs – hier könnten organisatorische Maßnahmen ansetzen.

Die Erfassung der AVEM-Muster mit deren unterschiedlichen Ausprägungen in den einzelnen Dimensionen dient dazu, wichtige Indikatoren psychischer Gesundheit der Einsatzkräfte darzustellen. In Hinblick auf die psychische Gesundheit der Einsatzkräfte und auf den bereits bestehenden Fachkräftemangel im RD sollten Organisationseinheiten sensibilisiert und aufgefordert werden, individuelle gesundheitsfördernde und präventive Maßnahmen anzubieten. Dabei ist der Beschäftigte darüber aufzuklären, dass nicht nur organisationsbezogene Belastungsfaktoren auftreten, sondern auch die Persönlichkeit und somit das persönlichkeitsspezifische Verhalten und Erleben entscheidend für die gesundheitliche Entwicklung während des Berufslebens sind [44]. Verhaltens- und Verhältnisprävention sollten einander ergänzen. Die AVEM-Muster A, B und S (s. Infobox 1) sollten unter dem Interventionsaspekt gesehen werden. Bei personenbezogenen Interventionen kann direkter auf den Verhaltens- und Erlebensbereich eingewirkt werden, um gesundheitsgefährdende Muster abzubauen und gesundheitsförderliche zu stärken.

Als Interventionsmaßnahmen bei innerer Unruhe und Unausgeglichenheit oder eingeschränkter Distanzierungsfähigkeit bieten sich Entspannungstechniken, Ausagieren durch Sport, Bewegung oder Gartenarbeit an. Außerdem erscheint hier auch ein Konflikt- und Stressbewältigungstraining empfehlenswert (v. a. für Muster A; [44]). Besteht ein eingeschränktes Lebensgefühl, ist die Schaffung von Zufriedenheitserlebnissen sinnvoll [44]. Fehlender Entspannungsfähigkeit kann mit individueller Stressanalyse sowie kurz- und langfristigem Stressbewältigungstraining entgegengewirkt werden [44]. Beim Erleben mangelnder sozialer Unterstützung sind die Entwicklung von Teamgeist und -fähigkeit oder die Schaffung von gutem Arbeitsklima ratsam [44]. Bei eingeschränkter kommunikativer Kompetenz oder defensiver Problembewältigung (v. a. bei Muster B) sind entsprechende Trainingsprogramme nützlich [44]. Resignation, Hoffnungslosigkeit oder Verzweiflung können ebenfalls mit Coaching in Einzel- oder Gruppensitzungen oder bspw. durch die Stärkung des Selbstbewusstseins aktiv entgegengewirkt werden [44].

### Limitationen

Die Aussagekraft der subjektiven Angaben der Befragten ist immer infrage zu stellen, da die Antwortverzerrung durch die soziale Erwünschtheit vorkommen kann. In der hier vorgestellten Arbeit wurden im Rahmen der Gefährdungsbeurteilung bzgl. Psyche die Belastungen, Ressourcen und (Fehl‑)Beanspruchungsfolgen deskriptiv analysiert. Erste Muster von Zusammenhängen wurden mittels Korrelationen geprüft, lassen jedoch keine kausale Interpretation zu.

## Fazit

In dieser Studie hat sich gezeigt, dass die psychische Widerstandskraft gegenüber Arbeitsbelastungen sowie die emotionale Einstellung gegenüber der Arbeit relevant sind, wenn es um eine ausreichende Erholung, um Beanspruchung oder gesundheitliche Beschwerden geht. Die Ergebnisse gehen mit anderen Studien konform. Die Erstellung einer Gefährdungsbeurteilung und die Erfassung vorhandener Ressourcen bzw. Merkmale des arbeitsbezogenen Verhaltens und Erlebens sind für die Erkennung möglicher Gesundheitsrisiken bei den hoch belasteten Einsatzkräfte im RD zu empfehlen. Sie können bei der Ausarbeitung von Vorschlägen zur Prävention und Gesundheitsförderung verwendet werden. Diese Studie leistet einen Beitrag zum Schließen der Forschungslücke auf dem Gebiet der Rettungsdienstforschung.

### Infobox 1 Arbeitsbezogene Verhaltens- und Erlebensmuster nach Schaarschmidt und Fischer (2008; [31])

Das *A‑Muster* beschreibt Personen mit übermäßigem *Arbeitsengagement* bei gleichzeitig verringerter Distanzierungsfähigkeit gegenüber den Arbeitsproblemen und einem negativen Lebensgefühl.

Beim *B‑Muster* geht man von einem *Burnout-ähnlichen Erscheinungsbild* aus: Die Distanzierungsfähigkeit ist erniedrigt, die emotionale Einstellung gegenüber der Arbeit ist negativ, die Motivation und somit das Arbeitsengagement sind abgeschwächt.

Das *S‑Muster* steht für *Schonung* und beschreibt einen Zustand mit geringerem Engagement, aber hoher Distanzierungsfähigkeit, psychischer Widerstandsfähigkeit gegenüber Belastungen und (relativer) Lebenszufriedenheit. Hier kann man von einem Motivationsproblem der Arbeit ausgehen [45].

Dem *G‑Muster* schreibt man *gesunde Einstellung* zu: deutliche, aber nicht exzessive Ausprägung im Arbeitsengagement und positives Lebensgefühl, in Kombination mit ausreichender Distanzierungsfähigkeit, offensivem Bewältigungsverhalten und psychischer Widerstandsfähigkeit. Insgesamt zeigen hier die Dimensionen für die emotionale Einstellung gegenüber der Arbeit die höchsten Ausprägungen im Vergleich zu den anderen Mustern und deuten somit auf ein positives Erleben hin.
